# Inositol 1,3,4,5-tetrakisphosphate: a remarkable second messenger in platelet signaling

**DOI:** 10.1016/j.rpth.2024.102365

**Published:** 2024-03-01

**Authors:** Elmina Mammadova-Bach, Attila Braun

**Affiliations:** 1 Walther Straub Institute of Pharmacology and Toxicology, Faculty of Medicine, Ludwig Maximilian University, Munich, Germany; 2 Division of Nephrology, Department of Medicine IV, Ludwig Maximilian University Hospital, Munich, Germany

In this issue of *RPTH* journal, Authi et al. [1] proposed an inhibitory function of inositol 1,3,4,5-tetrakisphosphate (IP4) in platelet activation using a selective inositol (1,4,5)-trisphosphate (IP3)-kinase inhibitor, GNF362.

Phospholipid metabolism plays a crucial role in platelet activation. Within a few seconds after platelet receptor stimuli, various forms of phosphatidylinositols (PIs) are produced and released from the plasma membrane into the cytoplasm, transmitting diverse signaling in the dense tubular system, mitochondria, secretory granules, and actin cytoskeleton. These metabolic changes are regulated by PI kinases and PI phosphatases, which phosphorylate or dephosphorylate hydroxyl groups of the inositol ring at different positions, resulting in 7 phosphorylated derivatives. Among these PIs, PI (4,5)-bisphosphate (PIP2) plays a central role in platelet signaling.

Platelet receptor–induced phospholipase C activation induces PIP2 hydrolysis, tightly regulating Ca^2+^ responses by mobilizing intracellular Ca^2+^ stores in the dense tubular system and activating Ca^2+^ channels in the plasma membrane. PIP2 hydrolysis serves as an important precursor of second messengers such as diacylglycerol and inositol (1,4,5) trisphosphate (IP3) [2]. These second messengers enhance platelet Ca^2+^ responses through the activation of the diacylglycerol-sensitive TRPC6 channel and IP3-receptor (IP3R)-mediated Ca^2+^ store depletion, respectively. The decreased Ca^2+^ level in the store activates stromal interaction molecule 1, which translocates to the plasma membrane, initiating store-operated Ca^2+^ entry (SOCE) by activating the ORAI1 channel [2]. Additionally, PIP2 is converted to PI (3,4,5)-trisphosphate (PIP3) by PI3-kinase (PI3K) during platelet activation. PIP3 recruits pleckstrin-homology (PH) domain–containing proteins from the cytoplasm to the plasma membrane, regulating downstream effectors such as protein kinase B (PKB)/AKT, Bruton’s tyrosine kinase (BTK), and the Ras and Rap GTPase activating protein 3 (RASA3), which was originally identified as an IP4-binding protein (GAP1^IP4BP^) in platelets [3,4].

In activated platelets, IP3 undergoes rapid phosphorylation to form inositol IP4 or dephosphorylation to form inositol 1,4-bisphosphate, thereby deactivating IP3R function and subsequent Ca^2+^ store depletion. The regulation of this process involves IP3 kinases (ITPK-A, ITPK-B, and ITPK-C), which phosphorylate IP3 to produce IP4 (Figure). Although the enzymatic activity of ITPKs and their metabolic products have been studied upon platelet activation [5,6], the role of IP4-mediated platelet signaling, particularly its involvement in thrombosis and hemostasis, has not been investigated.

**Figure:**
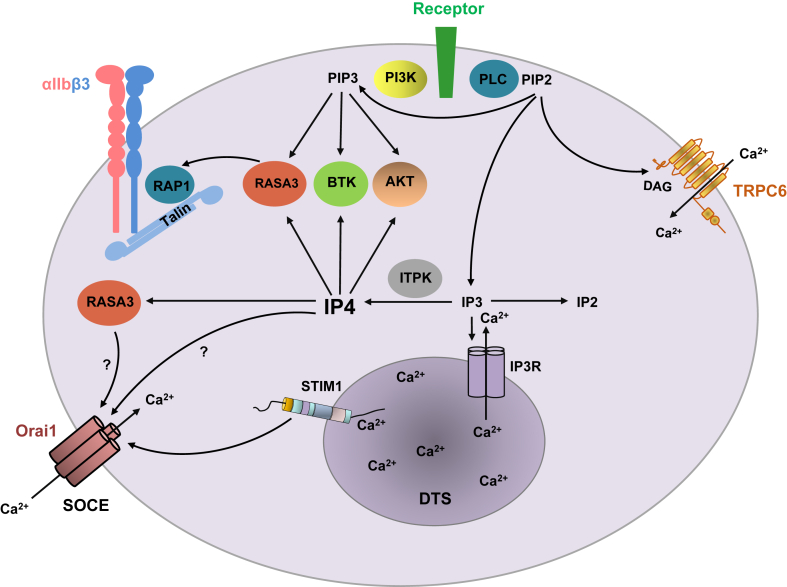
Proposed model of inositol 1,3,4,5-tetrakisphosphate (IP4)-mediated signaling in platelets. AKT, protein kinase B; BTK, Bruton’s tyrosine kinase; DAG, diacylglycerol; DTS, dense tubular system; IP2, inositol 1,4-bisphosphate; IP3R, (1,4,5)-trisphosphate-receptor; ITPK, IP3-kinases; PI, phosphatidylinositol; PIP2, PI (4,5)-bisphosphate; PI3K, PI3-kinase, PIP3, PI (3,4,5)-trisphosphate; PLC, platelet receptor–induced phospholipase C; RASA3, Ras and Rap GTPase activating protein 3; SOCE, store-operated Ca^2+^ entry; STIM1, stromal interaction molecule 1.

In mammalian cells, ITPK function is modulated by protein kinase C, protein kinase A, and Ca^2+^/calmodulin-activated protein kinase II [7]. The overexpression of ITPKs can rapidly convert IP3 to IP4, reducing the lifetime of IP3 and attenuating IP3-mediated Ca^2+^ responses [7]. Conversely, ITPK deficiency does not alter IP3 levels in many cell types [8], suggesting an alternative metabolic route for IP3. To date, only a limited number of IP4-binding proteins or downstream effectors have been identified, including ORAI1 channel and RASA3/GAP1^IP4BP^. Using patch clamp assay, it has been observed that increased levels of IP4 in the cytoplasm can rapidly block ORAI1 channel function [9]. Thapsigargin-induced SOCE was also inhibited by IP4 [10]. However, the exact molecular mechanism of channel desensitization remains elusive. It is still an open question whether IP4 might directly bind to the channel pore of ORAI1 or indirectly regulate the channel function through RASA3/GAP1^IP4BP^ signaling (Figure).

The tissue-specific distribution and functional roles of ITPKs have been studied in mammals. ITPK-A exhibits high expression in the central nervous system. In ITPK-A–deficient mice, enhanced long-term potentiation was observed in neurons [11]. ITPK-B is detected in hematopoietic cells, T and B cells, as well as neutrophils [12]. Deficiency of ITPK-B significantly reduces IP4 production in mouse thymocytes, also resulting in impairment in T-cell maturation and B-cell survival [8,13]. Abnormally increased SOCE was observed in ITPK-B–deficient B cells, probably due to the absence of ORAI1 channel desensitization through IP4-mediated signaling [9,14]. ITPK-B–deficient mice exhibit hyperactivated neutrophils, resulting from dysregulated phospholipid metabolism and consequent PIP3-mediated PKB/AKT response [15]. In various ITPK-B–deficient cells, enhanced PIP3-dependent membrane translocation of PKB/AKT and RASA3/GAP1^IP4BP^ was observed, suggesting that IP4 appears to compete with PIP3 by binding similar PH-domain–containing proteins, thereby blocking PIP3-induced protein trafficking. The expression profile of ITPK-C closely resembles that of ITPK-B, and ITPK-C–deficient mice are healthy; therefore, it was proposed that ITPK-B can compensate for the absence of ITPK-C functions *in vivo* [8,16]. Despite the generation of several mouse models emphasizing ITPK-dependent pathomechanisms, limited and controversial studies have linked human *ITPK* gene polymorphism or dysfunction to disorders such as Kawasaki syndrome [17], and no platelet results have been published.

In this issue of *RPTH* journal, Authi et al. [1] demonstrated that both ITPK-A and ITPK-B isoforms are expressed in platelets. The novel ITPK inhibitor GNF362 effectively inhibits both ITPK isoforms in platelets. At the threshold concentration of platelet agonists, including adenosine diphosphate, collagen, thrombin, and thromboxane A2 analogue U46619, aggregation responses were enhanced in the presence of GNF362 [1]. Using GNF362-treated whole blood under arterial shear stress conditions, the flow chamber assay showed increased thrombus formation on the collagen-coated surface *ex vivo*, suggesting an antithrombotic potential of ITPK-mediated signaling [1].

Platelets loaded with Ca^2+^ indicator Fura-2 displayed elevated Ca^2+^ responses after agonist stimuli in the presence of GNF362, correlating enhanced Ca^2+^ responses with reduced IP4 and slightly increased IP3 levels in the cytoplasm [1]. As IP4 is not a membrane-permeable compound, extracellular IP4 cannot penetrate into platelets. To overcome this technical difficulty, the authors used saponin-permeabilized platelets to further investigate IP4-mediated signaling [1]. Saponin-treated platelets were activated with a nonhydrolyzable analogue of guanosine triphosphate (GTPγS) or IP3 to accelerate platelet aggregation, and this response was diminished in IP4-treated platelets. Interestingly, GTPγS treatment induced Ser473 phosphorylation of AKT in platelets, and this effect was inhibited by IP4 or the PI3K inhibitor LY294002, indicating a functional crosstalk between IP4- and PI3K-mediated signaling [1].

RASA3/GAP1^IP4BP^ signaling inhibits Rap1 function and consequent α_IIb_β_3_ integrin-mediated platelet aggregation [18,19]. The authors proved this concept by demonstrating that GTPγS can induce Rap1 activation in saponin-permeabilized platelets, and this process was impaired in the presence of IP4. Interestingly, LY294002 did not affect GTPγS-induced Rap1 activation, indicating that the IP4/RASA3/GAP1^IP4BP^ signaling route regulates Rap1 activity independently of PI3K function.

Abolished ITPK function increases PIP3-mediated signaling in many cell types. The authors performed a PIP3 pull-down assay with platelet lysates and showed that RASA3/GAP1^IP4BP^ could effectively interact with PIP3. This interaction was inhibited with IP4 inclusion but not with IP3, highlighting the crucial role of IP4 in the regulation of RASA3/GAP1^IP4BP^ function. In agreement with previous findings in mammalian cells, the interaction between PIP3 and BTK was also inhibited in the presence of IP4 in the platelet lysates. Altogether, these results suggest that IP4 acts as a strong competitor of PIP3 by binding to similar PH-domain–containing proteins and modulating their functions in platelets.

The study by Authi et al. [1] has provided new insights into the regulatory function of IP4 in platelet signaling [1]. ITPK activity controls phospholipid metabolism in resting platelets, maintaining an equilibrium between phospholipid pools of IP3, PIP3, and IP4. Upon platelet activation, ITPK rapidly increases IP4 levels, thereby triggering antagonistic effects on IP3- and PIP3-mediated signaling, which could influence IP3R, RASA3/GAP1^IP4BP^, BTK, and AKT pathways (Figure). The development of cell-permeable mimetics of IP4 and the identification of novel ITPK agonists are crucial for elucidating the antithrombotic potential of IP4 and ITPK activity *in vivo*. Understanding the molecular mechanism of IP4-mediated regulation of ORAI1 function is an essential step in this research, given that the blockade of ORAI channel activity has therapeutic potential in arterial thrombosis and stroke [2,20]. Further studies in ITPK mouse models and human patients are necessary to investigate the link between dysregulated ITPK functions and abnormal IP4 levels in platelet-related diseases.
